# 2346: Receptor for advanced glycation end-products: Mitigating the persistent effects of particulate matter induced airway injury

**DOI:** 10.1017/cts.2017.42

**Published:** 2018-05-10

**Authors:** Syed Hissam Haider, Liqun Zhang, George Crowley, Erin J. Caraher, Rachel Lam, Sophia Kwon, Ann Marie Schmidt, Lung-Chi Chen, D. J. Prezant, Anna Nolan

**Affiliations:** 1 NYU School of Medicine, New York, NY, USA; 2 Fire Department of New York City, New York, NY, USA

## Abstract

OBJECTIVES/SPECIFIC AIMS: Obstructive lung disease following particulate matter (PM) exposure is a major health concern. Coexisting metabolic syndrome (MetSyn) often occurs. Receptor for advanced glycation end products (RAGE) is highly expressed in the lung, is a strong predictor of FEV1, and is a key mediator of MetSyn. To determine if the loss of RAGE protects from the persistence of effects of particulate associated lung injury in a murine model. METHODS/STUDY POPULATION: Wild type (WT) and RAGE knockout (RKO) mice were exposed to 100 μg of PM (WTC-Aggregate, PM53) or PBS control by oropharyngeal aspiration. Lung function, methacholine challenge, and bronchoalveolar lavage (BAL) were quantified 28 days after PM exposure using flexiVent (Scireq Montreal, QC). BAL was obtained and cell differentials, cytokines and transcription factors were assayed. Bio-volume to airspace ratio and mean chord length were measured (Image J and Adobe Photoshop). RESULTS/ANTICIPATED RESULTS: WT mice were hyper-reactive to methacholine compared with their PBS controls 28 days after a single exposure to PM. They recovered from increased neutrophilia, loss of FEV, decreased compliance, and increased resistance, which were previously observed 24-hours after exposure. RKO were not hyper-reactive when compared with their own PBS controls. Lung histology shows persistence of loss of alveolar space in WT mice exposed to PM after 28 days. Area fraction was significantly higher in PM exposed WT mice after 28 days which was not significant after 24 hours. Mean chord length was significantly shorter for PM exposed at both time points for WT mice. The relative expression of phosphorylated to total CREB and ERK1/2 proteins was lower in RKO PM exposed mice compared with WT PM while STAT3 expression was lower in WT PM compared with WT PBS. PM induced a lower fold change of total proteins from the PBS controls in RKO for CREB, p38, ERK1/2, STAT3, and STAT5. JNK and p70S6k total proteins expressed a decreased fold change in WT PM exposed mice compared with WT PBS controls. DISCUSSION/SIGNIFICANCE OF IMPACT: A single dose of PM can produce persistent airway hyper-reactivity after 28 days of exposure. This PM induced injury is alleviated in the absence of RAGE, similar to what was seen at 24 hours. Inhibiting RAGE may be key to limiting the persistent inflammatory effects of high intensity PM exposure.

